# Salmagundi

**Published:** 1887-11

**Authors:** 


### SALMAGUNDI.

EDITED BY E. G. BETTY, D.D.S.

THE case of a child has recently been reported who had four sets of teeth before the age of fifteen years was reached. The first set appeared at the age of six months. These teeth were all shed at nine months. At eleven months she began teething again; another set of teeth being erupted in four months. Six weeks after these teeth began to crumble and were entirely lost. Her weight at this time was ten pounds. At thirty months the third set appeared, and these remained until the age of four years, when they were extracted. The fourth set began to erupt at eleven years, and the dentition was completed at fifteen.—*Clipping.*

PAROTITIS AND PELVIC DISEASE.—Mr. Stephen Paget has collected no less than 101 cases of parotitis consequent upon injury or disease of the abdomen or pelvis. Of this number, 10 were due to injury or disease of the urinary tract; 18 were due to injury or disease of the alimentary canal; and 23 were due to injury or disease of the abdominal, the peritoneal, or the pelvic cellular tissue. The remaining 50 were due to injury or disease, or temporary derangement of the generative organs. Under the latter term are included slight blows on the testis, the introduction of a pessary, menstruation, and pregnancy. This form of parotitis appears to be non-pyæmic, to have no definite incubation stage, and to end very often in suppuration. Mr. Paget considers that the sequence of disease is due to influences acting through the nervous system.

---

THE INTERNATIONAL MEDICAL CONGRESS.—The REGISTER publishes its report of the Congress elsewhere and it is a good one. A few odds and ends picked up by Salmagundi may not prove uninteresting. One of the most interesting things was Dr. E. B. Call's demonstration of his method of making gold crowns. The gold he uses is an alloy composed of gold 94 parts, silver 3 parts, copper 3 parts. This he claims makes a metal that combines facility of working with a better resistance to mastication than pure gold alone and as a rule will not be oxidized by the fluids of the mouth. The plate he rolls to a thickness of No. 24 American gauge. For a bicuspid, for instance, a disc about the size of a silver dime is cut out of the plate. It is then annealed and swaged with a graded series of steel punches, each succeeding one being a trifle smaller than the one preceding it. This set of punches gives the crown its approximate shape, the second set is made to carry the gold through the counter die, which gives the crown very nearly its perfect shape. The contouring of the crown is completed with a pair of pliers especially adapted for the purpose. It is then cut the proper length and fitted to the root, after which it is polished up and mounted in the usual way with oxyphosphate. Dr. E. H. Angle, of Minneapolis, contributed a paper entitled a "New System of Regulation and Retention," his apparatus being a light but strong frame of gold to be adapted to any case; it in some respects embraces a few of the features of Patrick's System, at least it so appeared to the writer judging from such glympses as he could get of the models. Drs. T. E. Weeks and D. B. Freeman both exhibited clamps, the former a modification of the Hewitt, and the latter what he calls a douple loop, devised for carrying the rubber cloth beyond the gingival margin, so as to expose cavities at that point.

